# Bibliographical Notices

**Published:** 1842-01-22

**Authors:** 


### BIBLIOGRAPHICAL NOTICES.

*A Physiological and Pathological Inquiry concerning the physical characteristics of the teeth and gums, the salivary calculus, the lips and tongue, and the fluids of the mouth, together with their respective local and constitutional indications.* As read before the American Academy of Dental Surgeons, at their Second Annual Meeting, held at Philadelphia, August 11, 1841. By Chapin A. Harris, M. D., D. D. S., Professor of Practical Dentistry in the Baltimore College of Dental Surgery, &c. &c.; Baltimore; Armstrong & Berry, 1841. pp. 119, 8vo.

The whirl of occupation attendant on the winter session of our collegiate and clinical schools permits only a hasty glance at the contents of this little volume, bearing, as it does, on a speciality, conventionally, though improperly separated from the routine of medical instruction in this country. There is much information in the work calculated to as sist the student of surgery in prosecuting researches into the history of this department of his profession. We have no doubt that many valuable facts and still more valuable suggestions might be gleaned from its careful perusal, notwithstanding the existence of certain obvious physiological errors, and even discrepancies in its pages, due, it may be, rather to the condition of the science than to neglect on the part of the writer. The subject is as obscure as it is important, and we are happy to witness every attempt to transfer it from the dominion of empiricism to that of philosophy.

The volume is beautifully "gotten up," and, in this respect, is creditable alike to the author and publishers. We recommend it to the careful perusal of those in our profession who are fitted by proper theoretical study and practical experience to weigh its positions justly and critically. It is the production of one well versed in the opinions of others, and who has enjoyed ample opportunities of testing them.

---

*American Journal of Medical Sciences. Jan.* 1842.

In the American Journal for January, 1842, we find the following articles which may be termed strictly medical.

1. *Notes on the Scurvy, as it appeared on board the U. S. Frigate Columbia, in her cruize around the World,*—1838–'39–'40. By Edward Coale, M. D., U. S. Navy. 2. *On Scorbutus, which prevailed in the United States Army at Council Bluffs and St. Peters.* By Samuel Forry, M. D., of New York. 3. *Observations on the Rapidity of the Pulse of the Insane.* By Pliny Earle, M. D. 4. *Case of Yellow Fever, with Remarks.* By Thomas Stewardson, M. D., one of the Physicians to the Pennsylvania Hospital. 5. *Case of Tetanus, following a retained Placenta.* By D. Humphrey Storer, M. D., of Boston, Mass. 6. *On the Euphorbia Maculata.* By William Zollickoffer, M. D. 7. *Observations on Dysentery, and on the use of Astringents.* By Stepehn W. Williams, M. D. 8. *Catalepsy induced by Animal Magnetism.* By Blanchard Fosgate, M. D., of Auburn, New York.

In accordance with the plan which we have laid down for our future guidance, we shall notice each of these articles—some of them very briefly; while from those which offer facts that we believe will be interesting to our readers, we shall extract such parts as embrace the substance of all that is novel or interesting. We shall pursue the same method with the most important foreign journals, and thus place our readers in possession of interesting matter, which would otherwise cost them much trouble and expense.

*Case of Yellow Fever, with Remarks.* By THOMAS STEWARDSON, M. D., one of the Physicians to the Pensylvania Hospital.

This article, by Dr. Stewardson, contains a dissection of the body of a seaman, dead of yellow fever, who was taken ill on the 16th of September at sea, twenty-one days after leaving New Orleans. He entered the hospital on the 25th of September, and died on the evening of the 27th. When admitted he was jaundiced, but in so enfeebled a condition that he was unable to answer questions intelligibly, and was in that stage of acute disease in which the symptoms become indistinct. On examination, the viscera of the head and thorax offered nothing remarkable.

"*Abdomen.—Liver* of moderate size, not enlarged, of good firmness, the lower surface of both lobes of a bluish gray colour, the upper surface of a uniform dull mustard colour, or perhaps more nearly resembling that of powdered gamboge; the colour internally was *every where uniformly* the same as that of the upper surface; the surface of a section was smooth, and very dry, except where moistened by the blood from the veins, which was watery and pale; of a homogeneous appearance, with scarcely any trace of distinction between the acini and parenchyma; it was not greasy. The *gall bladder*, contracted and pale, contained about ℥ij. of viscid, brownish bile. *Spleen* about 4 inches long by 2½ wide, of its usual bluish colour externally; firm in mass; internally of a deep claret colour; a section looking granulated, owing to the white lines of the cut cells of the organ; its cohesion very marked, the finger penetrating it with difficulty. *Stomach* of medium size, containing from one to two ounces of a fluid rather more consistent than water, of a bluish black colour, with which the whole surface of the mucous membrane was more or less stained, being least so along the small curvature; besides this, in the great *cul-de-sac* the membrane was marked with a dull, red injection, in points; surface generally smooth, mamelonated along the middle of the great curvature on the anterior face of the organ; consistence of mucous coat slight in the great *cul-de-sac*, where it was also thin; elsewhere, rather thinner than usual, and a little softened. In the great *cul-de-sac* at the seat of the red injection, there were several superficial depressions, probably ulcerations, from half a line to a line in diameter. The mucous follicles about pylorus not notably enlarged."

The duodenum contained a portion of fluid similar to that contained in the stomach, but was in other respects healthy. In the upper part was a considerable injection of red points, like that of the stomach. Follicles of small intestine healthy. In the large intestine the orifices of the follicles were enlarged; and in the lower part the mucous coat was softened.

The following remarks by Dr. Stewardson, relative to the lesions observed in this case, and bilious remitting fever, are interesting. We hope, however, that our southern friends will illustrate the pathology of yellow fever under more favourable circumstances.

"*Remarks.* We are unfortunately but imperfectly acquainted with the symptoms under which the patient laboured during the greater part of the course of the disease, as he entered the hospital almost moribund. His attack appears to have commenced about three weeks after leaving New Orleans, where the yellow fever has prevailed to a great extent during the past season, and ten days before his admission into the hospital. Being practically unacquainted with the features of yellow fever, I hesitated as regards the character of the disease, which as well as that of the patient who had died on the passage, was considered by the mate as undoubted yellow fever. The expression of countenance seemed to be different from any I had ever before witnessed; the eye remarkably wild when the patient was much roused; there was an appearance of great distress, restlessness and epigastric tenderness, whilst at the same time neither this region nor the hypochondria were distended. After death the appearances observed were precisely those described by M. Louis, as characterising the yellow fever of Gibraltar. The liver was of the colour of gamboge, remarkably dry and anemic; scarcely any bile in the gall-bladder; the stomach evidently inflamed and containing a black fluid which was also found in the intestinal canal; the spleen nearly natural; the rest of the contents of the abdomen as well as the other organs presenting nothing remarkable excepting the yellow tinge of the aorta, &c. The lesion of the liver was very peculiar and striking, answering precisely to M. Louis's description of that observed at Gibraltar. The colour in the present instance was almost exactly that of powdered gamboge, with which a section of it made after it had macerated for a day or two, was compared in the presence of the class. Besides the alteration of colour, it should be recollected that the liver was very dry and anemic, and that scarcely any bile was found in the gall-bladder, so that the secretion of this fluid would seem to have been almost arrested. The inflammation of the stomach was far from being severe, and the amount of black matter contained in it and the intestinal canal was not great, entirely insufficient certainly to account for the fatal termination, which can only be explained by admitting some change, probably of the blood, inappreciable by the ordinary methods of investigation, but sufficient to compromise the functions of important organs. It is possible indeed that this change, supposing it to be one of the blood, may have been a consequence of the lesion of the liver, as recent investigation seems strongly to confirm the idea, that a principal function of this organ is, like that of the lungs, to purify the blood and thus render it more fit for the support of the vital action. Still, however, even on this supposition, the alteration of the circulating fluid must be considered as the immediate cause of death.

In conclusion let us note the similarities and discrepancies which exist between the post mortem appearances, observed by M. Louis in yellow fever, and those observed by me in remittent fever, and described in the April number of this journal for the past year. In both, the organs contained in the cavities of the chest and cranium were found either entirely healthy or the seat only of such secondary changes as are common to many acute affections, if we except the frequent occurrence in yellow fever of certain blackish spots or masses in the lungs, dependent in great measure upon the exhalation of blood into their tissue, and also the frequent destruction of the epidermis of the œsophagus in the same disease. In both, the liver was in every case the seat of a peculiar alteration, having certain common characters, but strikingly different in the two diseases. In both, the stomach was in the great majority of cases inflamed, whilst the remainder of the intestinal canal, the mesenteric glands and kidneys were healthy, or nearly so. On the other hand we find no less striking differences. Thus in yellow fever, the liver without much alteration of size or consistence, was yellow, anemic, with but little bile in the gall-bladder, whilst in remittent it was generally enlarged and flabby, and always of a dark colour more or less resembling bronze, with a gall-bladder for the most part fully distended. In yellow fever the stomach or some part of the intestinal canal mostly contained a fluid black matter which was absent in remittent. The spleen in yellow fever was healthy or nearly so, whilst in remittent it was the seat of extreme softening and enlargement. A consideration of these three points of difference seems to me to be of the last importance in determining the question of whether yellow and bilious remitting fevers are distinct diseases. That they are so is now perhaps the most generally received opinion, derived from a comparison not merely of the difference of symptoms but of the circumstances of their origin and prevalence, and if to these we add the differences in the post mortem appearances above mentioned, scarcely a doubt, I think, can be entertained but that this opinion is correct. The enlargement and softening of the spleen in the bilious remitting and other types of fever originating from marsh miasmata, is a prominent fact attested by most writers who have investigated the pathological appearances of disease in warm climates; and this fact alone is almost sufficient to convince us that yellow fever, in which the spleen rarely presents any considerable traces of disease, must be essentially distinct in its nature and origin. The very opposite conditions of the liver and of the biliary secretion are worthy of especial attention, in reference to the present question, especially as the increase of this secretion in the one disease, and its diminution in the other, are severally characteristic of them throughout their whole course, as shown by an appeal to symptoms. To these, however, I will not allude further at present, as I shall shortly have occasion to recur to them in the continuation of my observations upon remittent fever."

